# Single Cell RNA Sequencing Reveals the Pathogenesis of Aortic Dissection Caused by Hypertension and Marfan Syndrome

**DOI:** 10.3389/fcell.2022.880320

**Published:** 2022-06-21

**Authors:** Li Zhang, Zhihuang Qiu, Hui Zheng, Xi Yang, Jianqiang Ye, Jian He, Yumei Li, Liangwan Chen

**Affiliations:** ^1^ Department of Cardiac Surgery, Fujian Medical University Union Hospital, Fuzhou, China; ^2^ The Key Laboratory of Fujian Province Universities on Ion Channel and Signal Transduction in Cardiovascular Diseases, The School of Basic Medical Sciences, Fuzhou, China; ^3^ Fujian Center for Safety Evaluation of New Drug, Fujian Medical University, Fuzhou, China

**Keywords:** aortic dissection, single-cell RNA sequencing, cell-cell communication, cell heterogeneity, RNA velocity

## Abstract

Aortic dissection (AD) is mainly caused by hypertension and Marfan syndrome. However, it is unclear whether the cellular components and functions are different between the two causes. A total of 11 aortic samples were collected for single-cell RNA analysis and 20 clusters were disclosed, including VSMCs, fibroblasts, endothelial cells, T cells, B cells, monocytes, macrophages, mast cells, and neutrophils components. There were differences in cell subclusters and function between hypertension and Marfan patients. The cells also had different differentiations. Cellchat identified cell ligand–receptor interactions that were associated with hypertension and Marfan-induced AD involving SMC, fibroblast, mo-macrophages, and T-cell subsets. This study revealed the heterogeneity of cellular components and gene changes in hypertension and Marfan-induced AD. Through functional analysis and the changes in intercellular communication, the possible mechanisms of different causes of AD were explained from a new perspective, so we can better understand the occurrence and development of diseases.

## Introduction

Aortic dissection (AD) is a critical disease in clinical practice. Aortic wall distension resulting in dissection rupture and sudden death is due to the inability of the vascular wall to withstand high intracavitary pressure (Schlatmann and Becker, 1977; Robicsek and Thubrikar, 1994; Patel and Arora, 2008). AD mostly occurs in young and middle-aged men. Without timely surgical intervention, the mortality rate of AD is up to 80%. There are two main causes of AD in patients, hypertension and genetic defects such as Marfan syndrome (Gawinecka et al., 2017). Hypertension is mainly the increase in blood pressure and increased stress on the vascular walls, which eventually leads to the dissection of the vascular walls (Akutsu, 2019; Dong et al., 2019). Marfan syndrome is mainly caused by the FBN1 mutation, rupture of aortic elastic fibers, and accumulation of collagen proteins, resulting in thinning and tearing of vascular walls (Takeda et al., 2018; Fletcher et al., 2020). The main characteristic of AD is the degeneration and even apoptosis and necrosis of vascular smooth muscle cells (VSMCs) (Wu et al., 2013a). However, the pathogenesis of both causes of induced AD is still unclear.

VSMCs are the main cell components of aortic walls, which regulate blood pressure and blood flow distribution and maintain vascular homeostasis. Mature VSMCs showed a very low proliferation rate and were synthetic (Gianluca et al., 2017; Yang et al., 2020). Due to the strong phenotypic plasticity of VSMCs, impaired VSMCs undergo phenotypic transformation, thus playing an important role in cardiovascular diseases (Owens et al., 2004; Agne et al., 2018; Touyz et al., 2018; Liu and Gomez, 2019). However, the changes of other cell populations have been neglected, especially the cellular heterogeneity in AD, which is obscured. Currently, it has been found that cellular heterogeneity plays an important role in a variety of diseases, such as tumors (Ding et al., 2020; Zhou et al., 2020); atherosclerosis (Cochain et al., 2018; Williams et al., 2020) due to single-cell RNA sequencing technology, which has allowed people to better understand the importance of cellular components in normal and diseases. In this study, single-cell RNA sequencing was used to detect and analyze the cell heterogeneity of vascular tissue samples from normal, hypertension, and Marfan-induced AD patients. To compare and analyze the changes in cell composition and function changes of the two causes, aiming to find the microscopic mechanisms of different causes of diseases, so as to better understand the pathogenesis of AD development.

## Materials and Methods

### Single-Cell Dissociation

Single-cell RNA sequencing experiments were conducted by GENECHEM laboratories. Aortic tissue was surgically removed and stored in MACS tissue storage solution (Miltenyi Biotec) until treatment. The processing of tissue samples was described below. To put it simply, the sample was first washed with phosphate-buffered saline (PBS), chopped into small pieces (about 1 mm^3^) on ice, and enzymolized for 60 min with 10 U/mL collagenase I (Worthington) and 8U/mL DNase I (Worthington) stirring at 37°C. After digestion, the samples were sieved with a 70 μm cell filter and centrifuged at 300 g for 5 min. After supernatant removal, granular cells were suspended in an erythrocyte lysis buffer (Miltenyi Biotec) for erythrocyte lysis. After washing with PBS containing 0.04% BSA, the cell microspheres were suspended in PBS containing 0.04% BSA and re-filtered with a 40 μm cell filter. Isolated single cells were then stained with Calcein-AM (Thermo Fisher Scientific) and Draq7 (BD Biosciences) to assess viability. Single-cell suspensions were further enriched with the MACS dead cell removal kit (Miltenyi Biotech).

### Single-Cell RNA Sequencing

The BD Rhapsody system was designed to capture transcriptome information from single cells (from human samples). Single-cell capture was the random distribution of single-cell suspensions in >200,000 micropores by means of limited dilution. Beads with oligonucleotide barcodes were added to a saturated state to pair the beads with cells in the micropores. The cells were cleaved in micropores to hybridize mRNA molecules, and the oligonucleotides on the beads were captured by bar code. The beads were collected in a test tube for reverse transcription and ExoI digestion. During cDNA synthesis, each cDNA molecule was labeled with a unique molecular identifier (UMI) at the 5 ′end (i.e., the 3′ end of the mRNA transcript) and a cellular barcode indicating its cell origin. A BD Rhapsody single-cell whole transcriptome amplification (WTA) workflow consisting of random primer and extension (RPE), RPE amplified PCR, and WTA index PCR was used to prepare whole transcriptome libraries. The library was quantified using a highly sensitive DNA chip (Agilent) and Qubit highly sensitive DNA analysis (Thermo Fisher Scientific) on the Bioanalyzer 2200. Sequencing was performed by an Illumina sequencer (Illumina, San Diego, CA, United States) at the 150-bp end.

### Single-Cell RNA Statistical Analysis

We filtered adapter sequences using FASTP and default parameters and removed low-quality reads to get clean data. Single-cell transcriptome analysis was performed using UI tools to identify cell barcode whitelists. Clean UMI-based data were mapped to the human genome (Ensemble Version 91) using STAR mapping with parameters customized from UMI tools and standard pipes, to obtain UMI counts for each sample. For cells containing 200-6000 expressed genes, the rate of mitochondrial UMI was lower than 50%, and the mitochondrial genes were deleted from the expression table through cell mass filtration. Using the Seurat package (version: 3.1.4, https://satijalab.org/seurat/) according to each sample’s UMI count and the percentage rate of mitochondria, normalization and regression according to express table cells, scaling of the data. Because samples were batch processed and sequenced, samples were used to eliminate potential batch effects. In doing so, we create a potential anchor using the first 2000 variable genes and Seurat’s Find Integration Anchors feature. The data was then integrated using the integration data function to create a new matrix of 2000 features in which the potential batch effects were regressed. PCA was constructed based on large-scale data of the first 2000 high-variable genes, and tSNE and UMAP were constructed using the top 10 principal components. A graph-based clustering method (resolution = 0.8) was used to obtain unsupervised cell clustering results based on the first 10 principal components of PCA, and marker genes were calculated by using the Find All Markers function and the Wilcoxon rank-sum test under the criteria index, LnFC >0.25, *p* < 0.05, Min. PCT >0.1. In order to identify cell types in more detail, clusters of the same cell types were selected for re-tSNE analysis, graph-based clustering, and marker analysis.

### SCENIC Analysis

To assess the regulatory intensity of transcription factors, we used a Single-cell Regulatory Network Inference and Clustering (pySCENIC, V0.9.5) (Aibar et al., 2017) workflow using RcisTarget and GRNboost’s 20,000 motif databases.

### QuSAGE Analysis (Gene Enrichment Analysis)

To describe the relative activation of specific genes through QuSAGE (2.20.0) analysis, as previously described, “angiogenesis” and “fatty acid metabolism” pathway activation.

### Differential Gene Expression Analysis

The function Find Markers with Wilcoxon rank-sum was used to identify gene expression differences between samples. The criteria were as follows: LnFC >0.25, *p* < 0.05, Min. PCT >0.1.

### Co-Regulated Gene Analysis

To discover the gene co-regulatory network using the find_gene_modules function of Monocle 3 with the default parameters (Cao et al., 2019).

### Re-Clustering

In order to identify different cell subpopulations by cell identity and differentiation status, T cells, macrophages, SMCs, and fibroblasts were extracted and classified in the first unsupervised cluster analysis. The general method is the same as above, except that we use Harmony to correct the batch effect.

### RNA Velocity Analysis

Cell RNA velocity analysis was done based on the Velocyto program. Comments are read by concatenation and unconcatenation is first executed using the velocyto.py command line tools. Then, the velocity to R pipeline was used for downstream analysis.

### Cell Communication Analysis

To enable systematic analysis of intercellular communication pathways, we used CellChat to analyze the intensity of ligand and receptor action. CellChat identified the differentially expressed ligands and receptors in each cell population, calculated the probability of each interaction according to the law of mass interaction, then tested it by random permutations and constructed a weighted digraph to represent the communication network (Jin et al., 2021).

### Data Analyses

R (3.6.2) was used for all statistical analyses. Continuous variables were expressed as mean ± standard deviation (SD). Categorical variables were summarized as counts and percentages (%). The χ^2^ test was used to compare with categorical variables. Fisher’s exact test was used to evaluate differential proportional analysis (DPA) based on permutation. The *t*-test (parametric) and the Wilcoxon rank sum test (nonparametric) were used to analyze the differences in categorical variables among different groups. Univariate and multivariate logistic regression analyses were used to assess the risk factors for the diseases. *p* < 0.05 was considered statistically significant.

## Results

### Single-Cell RNA Sequence Analysis Discloses Heterogeneity in Aortic Dissection

Ascending aortic tissues were collected from 3 organ transplant patients, 5 hypertensive and 3 Marfan syndrome induced-AD patients. All the AD samples were obtained from patients with acute type A AD, and other aortic diseases (e.g., atherosclerosis) were excluded. The diagnosis of acute type A AD was based on contrast-enhanced computed tomography angiography (CTA) and echocardiography.

**FIGURE 1:**
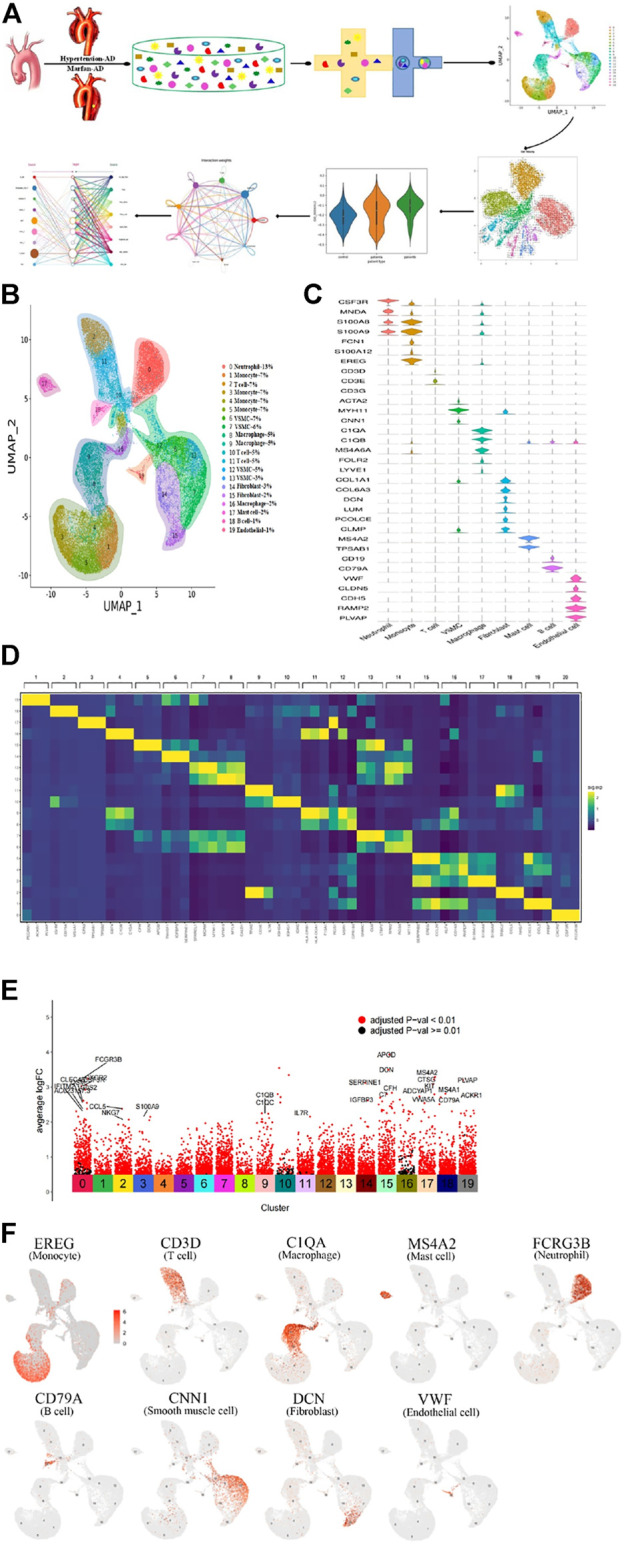
Single-cell RNA sequencing analysis reveals cellular heterogeneity in hypertension and Marfan-induced aortic dissection (AD). **(A)** The schematic figure for tissue collection, cell digestion, procession, isolation, and capture by the BD device, and then data analysis. **(B)** 20 distinct clusters of cells were identified and color-coded and are exhibited in the UMAP plot, showing the cellular heterogeneity. Different cell identities and percentages were defined for each cluster shown on the right. **(C)** The marker genes of different cell types are shown in the violin plot. **(D)** The top three genes and relative expression levels of each cluster are shown in the heatmap. **(E)** The significant upregulation genes in each cluster. **(F)** UMAP plots of expression distribution for selected cluster-specific marker genes. Mo, monocyte; Mac, macrophage; Neu, neutrophil; SMC, smooth muscle cell; Fib, fibroblast; EC, endothelial cell.

Marfan patients were diagnosed based on defined clinical criteria (Ghent nosology) and patients’ symptoms in Table 1. The patients’ detailed information is shown in Table 2. The tissues were placed in a cell storage solution and immediately sent to the company for enzymatic digestion into a single living cell suspension. Single-cell RNA sequencing was applied and analyzed, involving a single-tube protocol with unique transcript counting through barcoding with unique molecular identifiers (UMIs). The results of this study are shown in Figure 1A. The effective cells were obtained and screened through filtration, selection, and quality control as shown in Supplementary Figures S1A,B. Following gene expression normalization for read depth and mitochondrial read count, we applied principle component analysis on genes variably expressed across all 52,337 cells (details in Supplementary Figures S1C–E). We also observed the clustering analysis of each sample separately, as shown in Supplementary Figure S1F. PCA was applied to the samples in batches for integrated analysis.

**TABLE 1:** The diagnosis of Marfan syndrome (M) relies on defined clinical criteria (Ghent nosology).

Patients	With or without family history	With or without EL	Ao (Z-score)	Systemic score
M1	With	With	5	7
M2	With	With	4	9
M2	With	With	3	8

Ao, aortic diameter at the sinuses of Valsalva above indicated Z-score or aortic root dissection; EL, ectopia lentis.

**TABLE 2:** Patient information for aortic samples (*n* = 11).

Patient	Number	Sex	Age (years)	Diagnosis	Blood pressure	Smoking	Drink	Hypertension	Marfan	Other diseases
A1	hE062	M	50	Stanford A	172/80 mmHg	Yes	Occasional	Yes	No	No
A2	hE065	M	39	Stanford A	235/167 mmHg	Yes	Occasional	Yes	No	No
A3	hE066	M	35	Stanford A	154/73 mmHg	Yes	Occasional	Yes	No	No
A4	hE068	M	52	Stanford A	143/70 mmHg	Yes	Occasional	Yes	No	No
A5	hE075	M	56	Stanford A	145/82 mmHg	No	Occasional	Yes	No	No
M1	hE067	M	31	Marfan, Stanford A	114/70 mmHg	Yes	Occasional	No	Yes	No
M2	hE069	F	33	Marfan, Stanford A	144/47 mmHg	No	No	Yes	Yes	No
M3	hE098	M	51	Marfan, Stanford A	139/65 mmHg	Yes	Occasional	Yes	Yes	No
Con1	hE074	M	40	Receptor	92/59 mmHg	No	Occasional	No	No	Dilated heart disease
Con2	hE089	M	56	Receptor	94/70 mmHg	Yes	Occasional	Yes	No	CHD
Con3	hE118	M	37	Donor	103/74 mmHg	No	Occasional	Yes	No	Cerebral hemorrhage

Subsequently, we classified cells into groups of cell types using graph-based clustering on the informative principle components. Based on the known cell marker genes, we identified 20 clusters, including T cells, B cells, fibroblasts, endothelial cells; SMCs, neutrophils, macrophages, monocytes, and mast cells, representing nine main cell types (Figure 1B). The markers of each type of cell were shown in the violin plots (Figure 1C). The top three highly expressed genes in each cluster are displayed in the heatmap (Figure 1D), and the significant upregulation genes in 20 clusters were shown in Figure 1E. Each type of cell with a marker was colored in the UMAP plots, respectively,as shown in Figure 1F. Moreover, cell cluster genes and functional enrichment analysis results were supported in Supplementary Figures S2A,B.

### T-Cell Clusters in Aortic Dissection

**FIGURE 2:**
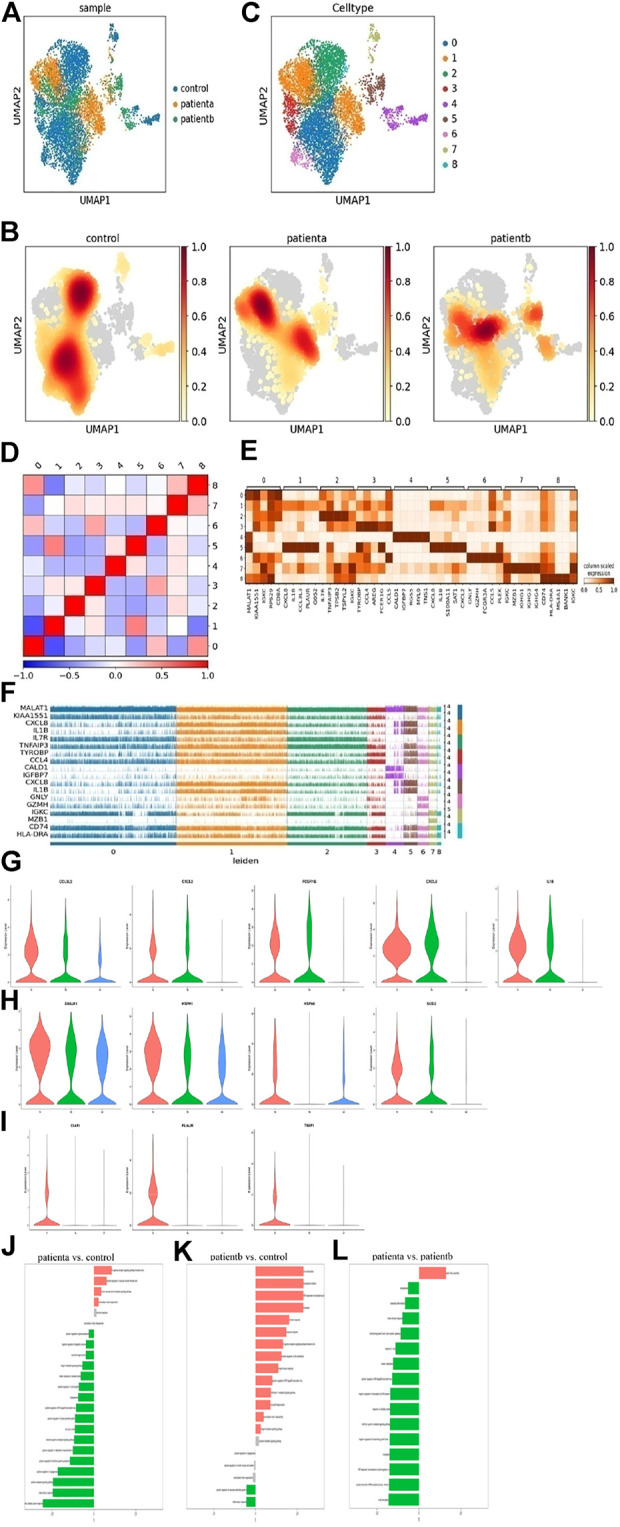
Heterogeneity of smooth muscle cells in human ascending thoracic aortic walls. **(A)** The UMAP plot shows the T cells of different samples by Seurat and Harmony analysis. **(B)** Nine subclusters of T cells were identified and color-coded are shown in the UMAP plot. **(C)** The nuclear density figure shows the different spatial distribution of T cells, the degree of red indicates the density of the cells. **(D)** Correlation analysis of T-cell subclusters. **(E)** Heatmap showing the top five genes and their relative expression in each subcluster of T cells. **(F)** The expression distribution of hypervariable genes in each cell. **(G)** The violin diagram shows elevated genes in T cells of diseases. **(H)** The violin diagram showed heat shock protein genes DNAJA1, HSPH1, HSPA6, and SOD2 expression increased in diseases. **(I)** The violin diagram shows a specific expression of disease a. **(J–L)** Differences n pathway activities scored per T cells by GSVA with disease a vs. normal **(J)**, disease b vs. normal **(K)**, disease a vs. disease b **(L)**. Disease a represents hypertension-induced AD, while disease b represents Marfan-induced AD. A: disease a, B: disease b, N: normal.

The changes of T cells in AD were analyzed, and T-cell clustering is shown in Figure 2A. The cell nuclear density map also showed differences in the spatial distribution of T cells between AD and normal samples. Meanwhile, it demonstrated that T cells were significantly reduced in hypertensive (disease a) and Marfan (disease b) samples, as shown in Figure 2B, indicating that the immunologic defense mechanism was weakened in AD. Furthermore, T cells were regrouped through Seurat, Harmony, and Scanpy, and we obtained nine subclusters (C0∼8) in T cells (Figure 2C). The sequence of highly variable genes in each cluster is shown in Supplementary Figure S3A.

The subclusters were defined based on the gene expression in each cluster. C0 was classified as a T helper and expressed CD8A, IL7R, TRBC2 and CCL5. C1 was named NKT, and expressed DNAJB1, CCL4, AREG, IL1B, and IFN. These cluster cells may play a role in anti-inflammation and ECM deposition, cholesterol transport, and phagocytosis. C2 was identified as a naive T cell with marker gene expressions, such as IL7R, TRBC2, and DGKA. C3 was classified as an exhausted CD8^+^ T cell with GNLY, CCL5, CCL4, and GZMH gene expression. C4 cells were enriched for SMC markers like RGS5, MYL9, and MYH11, while C5 cells were enriched for macrophage markers like S100A8, S100A9, and CD163. C6 was named Tem, with markers GNLY, CCL5, GZMH, and FCGR3A. C7 was enriched in B-cell markers IGKC, MZB1, and IGHG1, which may be mixed at the junction of the two populations. C8 cells were identified as inflammatory T cells and expressed CD74, MS4A1, and HLA-DRA. This cluster may protect the normal body from pathological injury.

In addition, the correlation analysis of cell subclusters is shown in Figure 2D. The top 5 genes and relative expression levels in each subcluster were displayed in the heatmap (Figure 2E). The top 20 genes in every subcluster are shown in Supplementary Figure S3B. The expressions of the hypervariable genes in each cell were shown in Figure 2F, indicating that there were differences in gene expression among the subclusters. The immune function of T cells was dominant in normal samples, while in diseases, CCL3L3, CXCL2, FCER1G, CXCL8, and IL1B were highly expressed (Figure 2G), indicating a certain pro-inflammatory effect of these cells. In disease a, heat shock protein genes DNAJA1, HSPH1, HSPA6, and SOD2 expression increased (Figure 2H), indicating oxidative stress response in T cells. Moreover, we found that C5AR1, PLAUR, and TIMP1 had specific expressions in disease (Figure 2I). These genes are associated with immune activation and cell apoptosis.

To further explore the difference in T-cell function between hypertension and Marfan-induced AD, GO enrichment (in Supplementary Figure S3C) and GSVA were used. The specific data of GSVA were supplied in Supplementary Tables S1–S3. Results in Figures 2J,K show that compared with the normal samples, the immune function of T cells was weakened, the extracellular matrix was recombined, and the tumor necrosis factor pathway was activated in disease a. Compared with normal samples, disease b showed enhanced T-cell inflammation and immune response and activated cytokine pathways that significantly promoted extracellular matrix degradation. Compared with disease b, disease a has stronger immune and inflammatory responses in disease b, stronger elastic fiber recombination in disease a, and weaker TGF-β pathway activity, suggesting that the sustained activation of TGF-β pathway was an important feature of disease b. Therefore, it could be seen that disease b strengthened the inflammatory reaction of blood vessels and was more likely to lead to the destruction of vessel structure and rupture.

### Smooth Muscle Cell Clusters in Aortic Dissection

**FIGURE 3:**
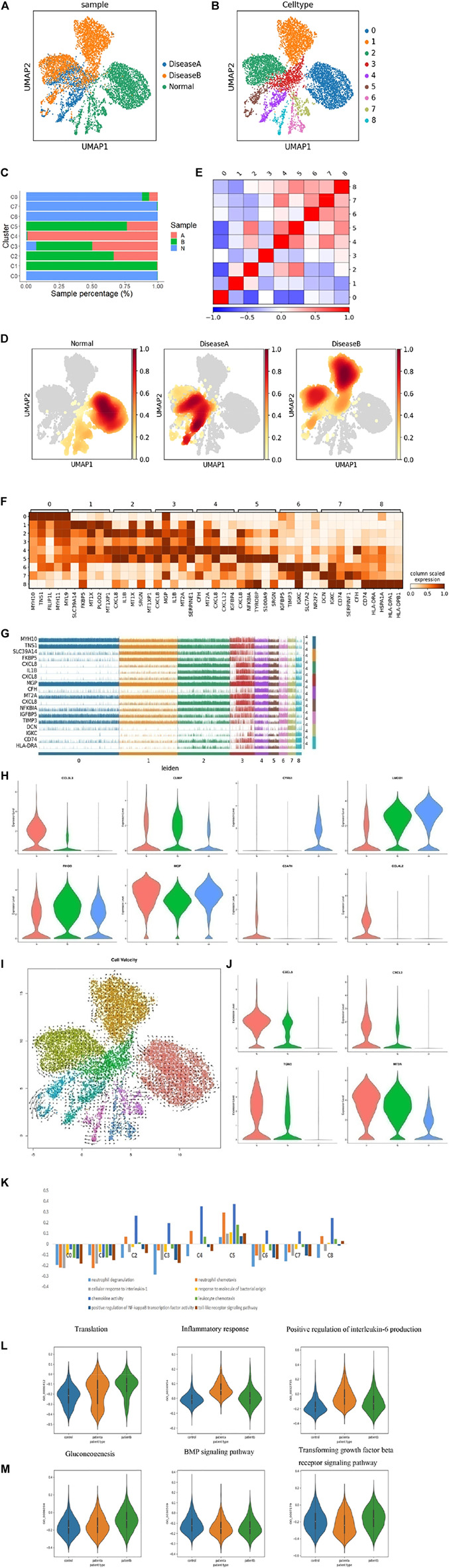
Heterogeneity of smooth muscle cells in human ascending thoracic aortic walls. **(A)** UMAP plot showing SMCs of different samples by Seurat and Harmony analysis. **(B)** SMCs were further divided into nine subclusters (C0∼8) shown in the color-coded UMAP plot. **(C)** The proportion of each cluster of cells in different samples. **(D)** Nuclear density figure showing the different spatial distribution of SMCs, the degree of red indicates the density of the cells. **(E)** Correlation analysis of SMC subclusters. **(F)** Heatmap showing the top five genes and their relative expression in each subcluster of SMCs. **(G)** The expression distribution of hypervariable genes in each cell. **(H)** The violin diagram shows some special changed genes in SMCs of diseases. **(I)** RNA velocity analysis showed the possible transition between different cluster cells. The arrow shows the direction of the transformation. **(J)** The violin diagram showing some special changed genes in subcluster SMCs of diseases. **(K)** Differences in pathway activities scored per SMCs by GSVA with different subclusters. **(L,M)** Differences in pathway activities scored per SMCs by GSVA among disease a and normal **(L)**, disease b and normal **(M)**. A: disease a, B: disease b, N: normal.

Smooth muscle cells (SMCs) are the core components of aortic vessels and play a key role in the pathogenesis of AD. Clustering analysis of SMCs in 11 samples was performed (Figure 3A), and then subclusters were obtained for C0∼8 subgroups (Figure 3B). The expression genes were sequenced, as shown in Supplementary Figure S4A. The top 20 genes in each subcluster are also shown in Supplementary Figure S4B. As we can see from Figure 3C, the number of SMCs was significantly reduced in diseases, especially in disease a. Nuclear density analysis also showed a decrease in the number of SMCs in AD patients (Figure 3D). AD is known to be characterized by SMC degeneration and apoptosis. Disease b was characterized by the destruction of elastic fiber tissues and collagen deposition by gene mutation, and disease a was characterized by pathological changes of SMCs and endothelial cells under long-term mechanical pressure, both of which eventually led to the reduction of SMC numbers and vascular rupture. A correlation analysis of SMC subgroups was performed, as shown in Figure 3E.

Next, we defined subsets of SMCs based on known markers and gene expression. C0 was defined as contractile SMCs, with MYH11 and RGS5 expression, and maintained a normal vasoconstriction state. C1 was defined as contractility, expressing MYH11, TNS1, and RGS5, as well. It also expressed ADAMTS1, VCAN, and TNS1 genes, which regulated the extracellular matrix. C2 was named “contractility,” expressing MYH11, MYL9, and RGS5. Meanwhile, these cluster cells also expressed CXCL8, IL1B, CXCL2, CCL3L3, NFKBIA, CXCL3, CCL20, and CCL4 genes, indicating that these cells secreted and released chemokines to regulate cytokine activity and recruit inflammatory cells. C3 was considered to be a macrophage type that expressed CXCL8, SPP1, CD74, IL1B, and toll receptors. And these cells could regulate collagen and inflammatory responses because TNC, VCAN, and FN1 genes were also highly expressed in these cluster cells. C4 was considered as endothelium-like SMCs, which expressed CXCL12, IGFBP4, PDK4, and collagen relative genes MMP2, COL1A1, 2, and TIMP1, indicating that this group of cells could migrate and lead to endothelial remodeling and angiogenesis. C5 was defined as a macrophage type, expressing CXCL8, S100A9, S100A8, and CD163, indicating inflammatory response activation in this cluster cells. C6 was named epithelial SMC with IGFBP4, PDK4, LGI4, and Notch3 expressions, suggesting cells might have differentiation potential. C7 was considered fibroblasts with expressed DCN, LUM, MMP2, and TIMP3, and these cells also highly expressed collagen genes COL1A1, COL3A1, and COL6A3, suggesting cells could regulate the collagen and extracellular matrix, and maintain normal vascular structures. C8 was also defined as macrophage SMCs that expressed C1QB, C1QA, C1QC, and HLA-DPA1. Its main function was to resist viruses and it has anti-inflammatory and defensive effects. Based on the analysis of cell composition and functional differences among each subcluster, we found that normal SMCs were dominated by the contractile type. In diseases, the cells still express contractile types, but their functions tend to regulate the matrix and secrete collagen. In addition, some contractile SMCs transformed into macrophage-like SMCs, which had a pro-inflammatory effect, and that in disease b was more prominent. In addition, disease a could switch into endothelium-like SMCs, which may be involved in angiogenesis. Through the GO enrichment analysis, the functions of the subclusters are shown in Supplementary Figure S4C, which were consistent with the defined results.

In addition, the top 5 genes of each cluster and their relative expressions in each cell are shown in Figures 3F,G, respectively. We discovered some important genes changed in diseases. CCL3L3, CLMP, CTSL, NFKBIA, and PLAUR genes were upregulated in both diseases a and b, especially in disease a (Figure 3H, and Supplementary Figure S4D). These genes are associated with inflammation. Meanwhile, some genes like CYR61, LMOD1, TNS1, and ITGA8 were lowered in diseases (Figure 3H, and Supplementary Figure S4D), which were important in maintaining vascular integrity. Some genes were significantly different expression in disease a and b. RHOB was decreased in disease a, but rose in b, while MGP increased in b (Figure 3H). RHOB owns various cellular functions, such as cell proliferation and apoptosis. MGP depressed vascular calcification. Meanwhile, we found that C5AR1, CCL4L2, CCL4, CCL20, CD163, CXCL3, and CXCL12 were only expressed in disease a (Figure 3H and Supplementary Figure S4D), suggesting the recruitment of inflammatory factors and promotion of the inflammatory response were both more obvious in hypertension-induced AD.

To better understand the differentiation of SMCs in diseases, we performed RNA velocity analysis (Figure 3I) and found differences in the differentiation direction of SMCs between samples. Normal SMCs have the potential to differentiate into other cells. Disease a was actively differentiated, while disease b was relatively less differentiated. The possible reasons were that in the early stages of the diseases, hypertension directly caused continuous pressure on SMCs, leading to the differentiation of SMCs. However, Marfan diseases initially destroyed collagen and elastic fibers and damaged the stent structure of blood vessels, resulting in the changes in SMCs (Schlatmann and Becker, 1977; Pyeritz and McKusick, 1979; Hollister et al., 1990; Hirata et al., 1991; Robicsek and Thubrikar, 1994). MYH11 and RSG5 were highly expressed in normal SMC samples, which were the key factors in contractile SMCs. CXCL8, CXCL2, TGM2, and MT2A genes were highly expressed in diseases (Figure 3J), which were mainly related to collagen and inflammatory response. It indicated that SMCs tended to differentiate into macrophages in diseases, which secreted inflammatory factors, degraded collagen, and promoted elastic fiber fracture and vessel rupture. This effect was more robust in SMCs of disease a. The GO pathway enrichment analysis data showed that the inflammatory pathway was the most prominent, especially in diseases (Supplementary Figure S4C), which was consistent with the previous results.

We performed GSVA, in order to compare the functions of SMC subclusters. The results indicated that there were different functions in the SMC subgroups. Among them, the pro-inflammatory response was the most significant change in the C5 subgroup. C5 was mainly the altered subgroup of disease a and b, especially in disease b (Figure 3K and Supplementary Figure S4E), indicating that inflammatory response was an important mechanism in the pathogenesis of AD. We further explored the differences between diseases a and b using GSVA. The results showed that inflammatory response and IL-6 production were significant changes in disease compared with normal, which was consistent with the clinical study that found a significant increase in serum IL-6 in AD patient serum. BMP and TGF-β pathways were significant changes in disease b compared to normal. We knew that disease b was mainly characterized by FN1 gene mutation, which led to sustained activation of TGF-β and caused collagen and elastic fiber degradation and, finally, vascular structure destruction. There were also differences between diseases a and b. Disease a, the IL-6 production and inflammation reaction, was more prominent (Figures 3L,M).

### Fibroblasts Clusters in Aortic Dissection

**FIGURE 4:**
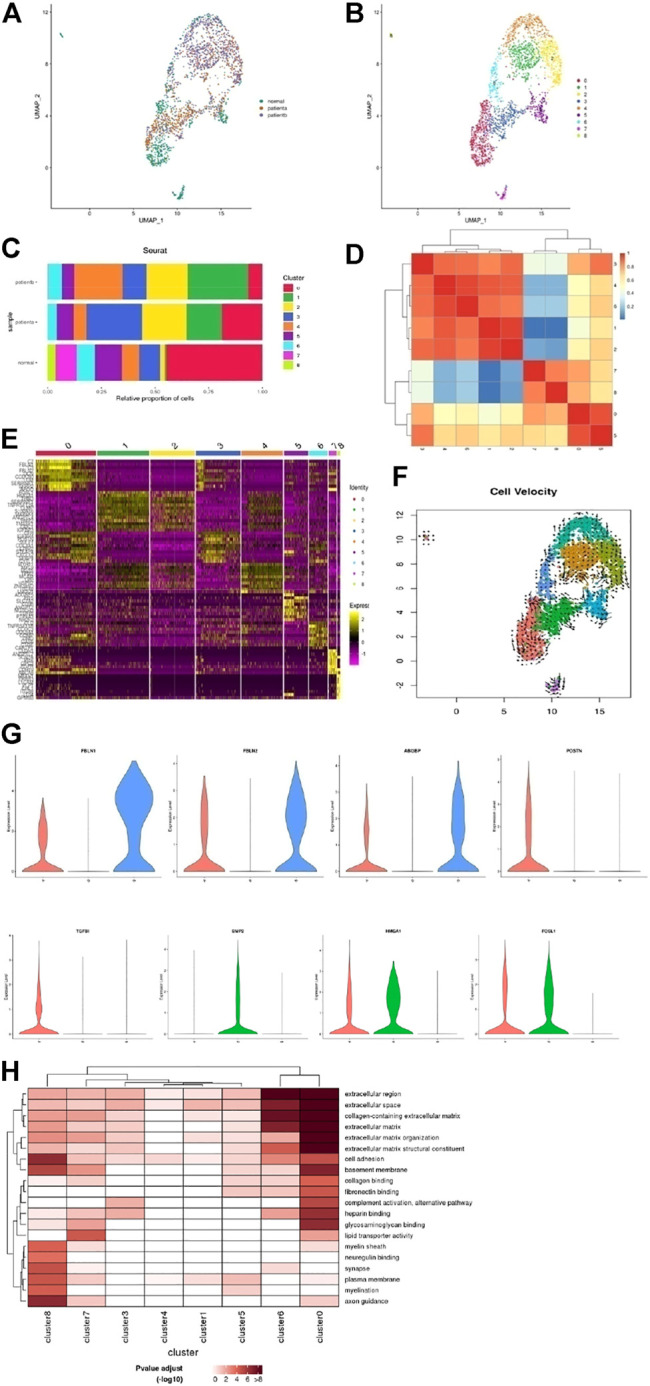
Heterogeneity of fibroblast cells in human ascending thoracic aortic walls. **(A)** UMAP plot of fibroblast cells colored according to identified clusters. **(B)** Nine subclusters of T cells were identified and color-coded as shown in the UMAP plot. **(C)** The cell composition proportion of fibroblast subclusters. **(D)** Correlation analysis of T cell subclusters. **(E)** Heatmap showing the top five genes and their relative expression in each sub-cluster of fibroblast cells. **(F)** The RNA velocity analysis shows the possible transition between different cluster cells. The arrow shows the direction of transformation. **(G)** The violin diagram shows some special changed genes in subclusters of diseases. **(H)** The significantly changed pathways in each subcluster were shown with GO enrichment analysis. A: disease a, B: disease b, N: normal.

Fibroblasts were clustered and reclustered from all the samples as well (Figures 4A,B). Based on the known markers and differentially expressed genes, we defined fibroblast subsets. C0 was defined as fibroblasts with DCN and LUM expression. C1 was named endothelium-like cells, as it expressed THBD, S100A16, and BMP2. C2 was classified as SMCs, with RGS5, MYH11, and MYLK gene expression. Other genes displayed inflammatory and chemokine expression (IL1B, CXCL8, CCL2, and CXCL2), suggesting fibroblast converted to SMCs and showed pro-inflammatory responses. C3 was defined as fibroblasts expressing LUM. These cluster cells expressed a large number of collagen genes. C4 was classified as SMCs and also expressed MYH11, MYLK, and RGS5. These cluster cells were associated with the recombination of elastic fibers that regulated cell migration and angiogenesis. C5 was named SMCs with Notch3 expression, which suggested the cells may be from nerve development. C6 was classified as matrix fibroblasts and expressed ELN. C7 was defined as fibroblasts with DCN and LUM expressed. In addition, ABCA genes (ABCA8,6,9,10) were highly expressed in these cluster cells, which transported cholesterol and nutrients. C8 was named neural progenitor cells, composed of vascular adventitia, and these have the possibility of differentiation. Moreover, the significant genes in each cluster are shown as UMAP and violin plots in Supplementary Figures S5A,B. Thus, fibroblasts maintained the normal structure of the extravascular matrix. They would transform into SMCs or endothelial cells in diseases, contributing to the vessel remodeling in AD.

The proportion of fibroblasts in different samples is shown in Figure 4C. We found that the proportion of fibroblast subsets underwent significant changes among samples. C0 was mainly in the normal vessels, while it was reduced in diseases, especially in disease b. C1 was increased in diseases a and b, more apparent in disease b. The BMP pathway and inflammation reaction were activated in these cluster cells through epithelial-mesenchymal transition and regulated angiogenesis, which suggested disease b affected the intima of the adventitia. C2 was increased in both diseases a and b, the common differentiation from fibroblasts to SMCs, secreting cytokines that promoted inflammation. C3 was mainly in disease a, regulated ECM. C4 was increased in disease b. C5 was reduced in disease b more than a. These cells affected lipid metabolism. C6 was reduced in disease a more than b, blocked cell–cell junction. C7∼8 were weakened in diseases, which showed that the effect of material transport was weakened in diseases. Thus, fibroblasts also played an important role in AD, and the mechanism of hypertension and Marfan-induced AD were somewhat different. A correlation analysis of each cluster is shown in Figure 4D. The top 10 genes of each subgroup were shown in the heatmap (Figure 4E).

RNA velocity analysis revealed the possibility of fibroblast differentiation (Figure 4F). Fibroblasts and neural progenitor cells in normal samples were more likely to differentiate. As Figure 4G shows, FBLN1/2 was required to maintain vascular structure, and the increase in FBLN1/2 could reduce MMP2/9 and also oxidative stress damage, which was beneficial in increasing vascular resistance to the risk factors. Abnormal expression of ABI3BP is associated with aging. These genes were reduced in both diseases a and b, respectively, in disease b. Diseases a and b were able to transition into SMCs, but the functions were different. In disease a, POSTN/TGFBI was highly expressed. POSTN-induced by TGF-β1 in fibroblasts led to the migration and invasion of ovarian cancer cells. Disease b expressed BMP2, which regulated angiogenesis and MMP-9 secretion. HMGA1 and FOSL1, which promote inflammatory response genes, were upregulated in both diseases, suggesting these cells like to differentiate into other cells. The results of the GO enrichment analysis also showed the functional differences of each subset, as shown in Figure 4H.

### Monocytes and Macrophage Clusters in Aortic Dissection

**FIGURE 5:**
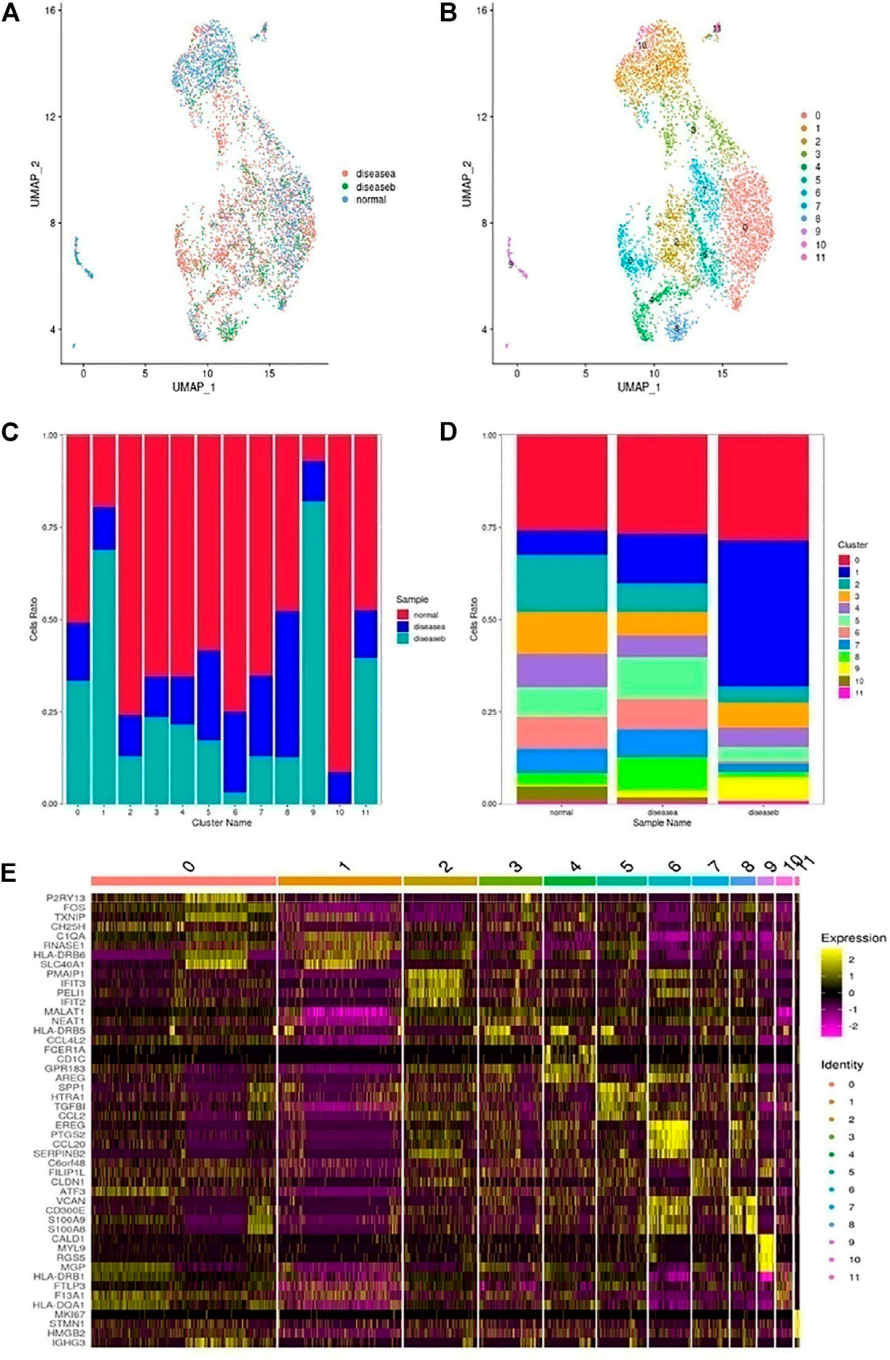
Heterogeneity of monocytes and macrophages in human ascending thoracic aortic walls. **(A)** UMAP plot of monocyte and macrophage cells colored according to identified clusters. **(B)** Monocytes and macrophages were further divided into 12 subclusters (C0∼11) as shown in the color-coded UMAP plot. **(C)** The plot shows the proportion of cells in each sample. **(D)** The plot shows the composition of cell subclusters. **(E)** The heatmap shows the top five genes and their relative expression in each subcluster of monocytes and macrophages. A: disease a, B: disease b, N: normal.

The monocytes and macrophages were reclustered from all samples in the UMAP plot and we got 12 subclusters (Figures 5A,B). Three subclusters (C2,6,8) were identified as M_remodeling clusters, expressing TNF, IL1B, NFKB1, EREG, AREG, and VCAN. C0 was defined as M2, expressing CD209, MRC1, MERTK, CD163, and NR4A3 genes. C1 was classified as M1, expressing C1QA, C1QB, and SOCS3. C3 was CD1C-CD141- dendritic cell with CCL4L2, CCL4, and CXCL8 highly expression. C4 was defined as cytotoxic CD8^+^ and expressed C1QA, C1QB, RUNX3, HLA-DQA1, and CXCR4. C5 was M1, expressed cytokines, and promoted inflammation with CCL4L2 and CCL4. C7 was made of monocytes, had T-cell properties, regulated cell growth, and expressed immune genes. C9 cells expressed MYH11, MYLK, and RGS5, suggesting switched SMCs and abnormal secretion of collagen and elastic fiber, aggravating and promoting AD occurrence. C10 was M2, with a high level of HLA-DQA1 family member expression. C11 were proliferation cells that expressed MKI67 and BIRC5.

From the cell composition ratio of macrophages in Figures 5C,D, we could see that there were more macrophages in normal samples than in disease samples, especially in disease a. It was speculated that the inflammatory response of disease a might be caused by other cells such as SMCs, fibroblasts, and T cells. DC (C3), CD8^+^ cytotoxic T (C4) and M2 (C0,10) were the main types of macrophages decreased in diseases. These cells were primarily protective. The increased subclusters were mainly C1/5/8/9, namely, M1 and SMC types in disease a. But in disease b, the increase of C1/9 was more obvious, which was related to the destruction of elastic fibers in disease b. The switched macrophages into SMCs mainly increased the secretion of cytokines, degraded collagen, and damaged elastic fibers, which aggravated the disease b development. The proportion of C5 in disease an increased while that in disease b decreased, indicating that disease a was mainly manifested by the inflammatory response of macrophages and the process of tissue remodeling, while disease b was more marked by the destruction of fibrous tissues and collagen changes. In addition, the top 10 genes of each subgroup are shown in the heatmap plot in Figure 5E.

### Cell–Cell Communication

**FIGURE 6:**
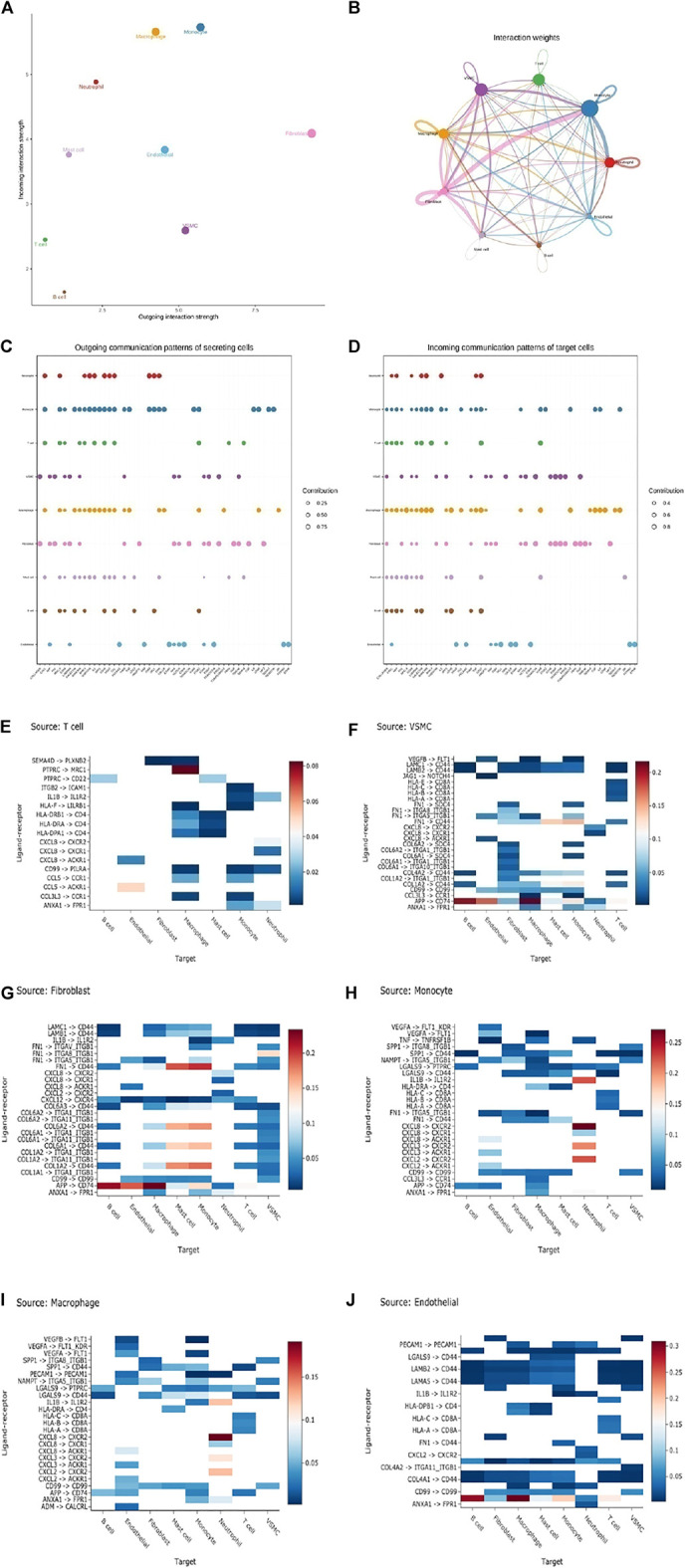
Cell-cell ligand-receptor network analysis. **(A)** Comparison of total incoming path weights with total outgoing path weights among all cell populations. **(B)** Circular network diagram of important cell–cell interaction pathways. Arrow and edge color represent direction (ligand and receptor), while edge thickness represents the sum of weighted paths among populations. **(C)** The dot plot shows the key ligands in the outgoing signal mode of the subset of secretory cells. **(D)** The dot plot shows the key receptors in the incoming signal mode of the subset of receiver cells. **(E–J)** The weight of the interaction shown in the heatmap was calculated as the product of the mean ligand expression of the source cell types, including T cells **(E)**, SMC **(F)**, fibroblast **(G)**, monocyte **(H)**, macrophage **(I)**, and endothelial cells **(J)** to the mean receptor expression of target cell types. (unilateral Wilcoxon rank-sum test and Benjamini–Hochberg false detection rate [FDR]> 0.01).

To confirm the potential intercellular interactions, we examined ligand and receptor relationships between different cell types in aortic vessels during AD progression. Based on a permutation test of random network connections consisting of weighted edges that reflect the changes in expression folding of ligands and receptors in the source and target populations described above, the number and weight of various cells in significant inbound connections are shown in Figures 6A–D. Next, we scored the interaction by calculating the average expression levels of receptors and ligands in each of the above cell types. The Wilcoxon rank-sum test was used to assess the statistical significance of each interaction score, and Benjamini–Hochberg multiple hypothesis correction was performed. Many interacting ligand-receptors were detected in T cells, SMCs, fibroblasts and Mo-Mac, associated with cell communication (Figures 6E–J).

**FIGURE 7:**
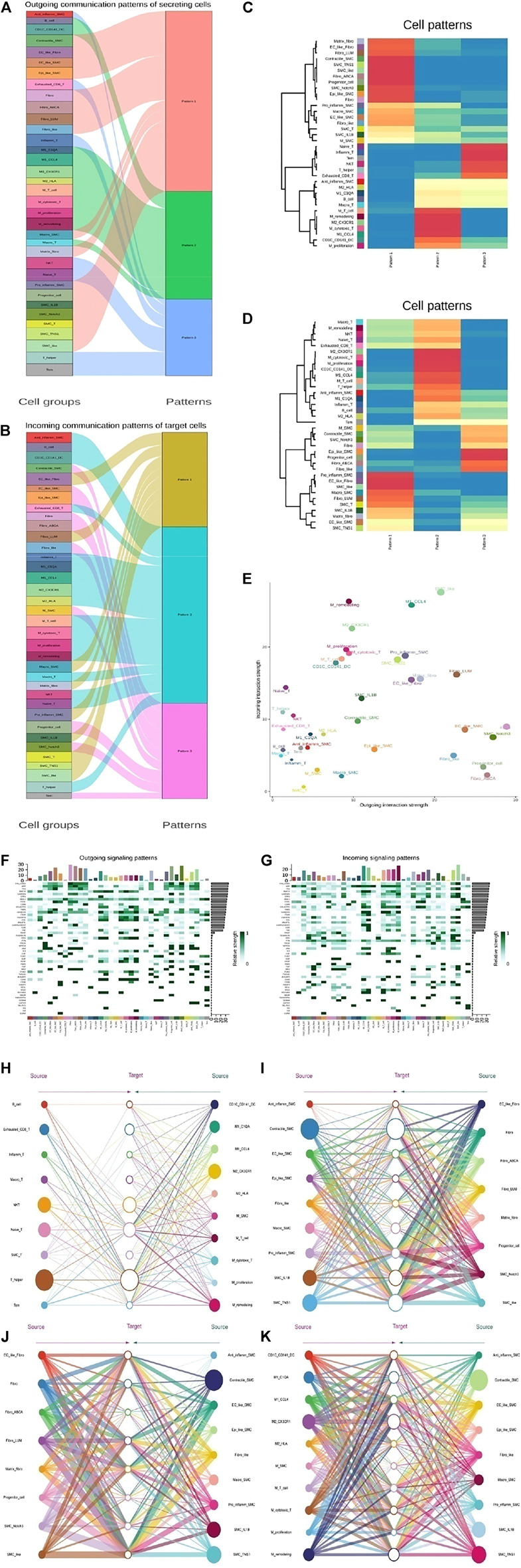
The key communications among four types of cell subpopulation. **(A)** River map showing the outgoing communication patterns of target cells by the key signals for subpopulations among T, SMC, fibroblast, and Mo-Mac cells. **(B)** River map showing the incoming communication patterns of target cells by the key signals for subpopulations among T, SMC, fibroblast, and Mo-Mac cells. **(C)** Heatmap showing the outgoing three communication patterns calculated by the key signals for subpopulations of target cells. **(D)** Heatmap showing the incoming three communication patterns calculated by the key signals for subpopulations of target cells. **(E)** Comparison of total incoming path weights with total outgoing path weights among T, SMC, fibroblast, and Mo-Mac cell populations. **(F)** Heatmap showing the key ligands in the outgoing signaling pattern of a subpopulation as secreting cells. **(G)** Heatmap showing the key receptors in the incoming signaling pattern of a subpopulation as receiver cells. **(H–K)** Hierarchical network diagram of important cell-cell communication modes between Tcell_vs._Mo-Mac **(H)**, SMC_vs._Fibroblast **(I)**, Fibroblast_vs._SMC **(J)** and Mo-Mac_vs._SMC **(K)**. Edge thickness represents the sum of key signals of weight between subgroups.

To further explore the detailed interactions of T cells, fibroblasts, VSMCs, and Mo-Mac, we first annotated each cell subtype with markers. Next, we used CellChat to further infer all possible communications within the cell subpopulations. Thus, each cell has a higher chance of having a weighted path for cell relationships than expected (P adj < 0.01). Hierarchical clustering showed that each cell subpopulation was completely divided into different groups, similar to three modes of interaction (Figures 7A–D and Supplementary Figures S7A–D). T cells, SMCs, fibroblasts, and Mo-Mac could all act as outgoings and incomings from the perspective of two kinds of intercellular interactions. Fibroblasts were the most effective as emitters, while T cells were the weakest. Specific analysis found that when T was outgoing, it was stronger mainly in the normal samples, which was to enhance the organism’s immunity. Among them, the SMC-T subgroup sent the strongest signal, but the signal of the receiver was the weakest, mainly in disease b. Under normal conditions, fibroblasts and macrophages output signals strongly to T cells while disease weakens them. SMCs affected T cells mainly strengthened in diseases, leading to an improvement in the immune microenvironment. Fibroblasts sent signals both in normal and in diseases, and as incoming, they became stronger in diseases. As signal outgoings, they responded significantly to macrophages, especially in disease a, indicating that they promoted inflammatory responses. In diseases, the signals of SMC receiving cells were enhanced, but the inflammatory SMC subgroup was weak, and the outgoing of macrophages was increased in diseases, promoting the inflammatory response of SMCs. The effect of SMC-T on SMCs also increased the inflammatory response of SMCs, mainly in disease b, indicating that under diseases, fibroblasts promoted the changes of SMCs. Macrophage signals were strong in normal and disease states, indicating inflammation throughout the entire disease process (Figures 7E–K and Supplementary Figures S6E–L).

## Discussion

This study revealed the main cellular composition of the ascending aorta in patients with AD due to hypertension and Marfan syndrome. It showed gene expression and changes in different cell populations. First, the cellular heterogeneity and functional changes of two pathogenic factors were explained from the cellular perspective. It was also found that macrophages communicated with immune cells and tissue cells, as well as between tissue cells, which played an important role in the occurrence of AD.

There were few studies on vascular cell heterogeneity until the advent of single-cell sequencing technology, which could identify the cell types and subcellular types of tissues and organs and even recognize the existence of new subsets of some diseases (Xie et al., 2018; Peng et al., 2019; Kalucka et al., 2020; Kinker et al., 2020). In mice, the heterogeneity of healthy vascular cells has been reported. ECs, SMCs, arterial fibroblasts, and immune cells, as well as a small neuronal cluster, have been identified (Dobnikar et al., 2018). The importance of vascular cell plasticity in diseases has also been recognized, as in atherosclerosis, where immune cells have been reported (Fernandez et al., 2019; Wolf and Ley, 2019). The composed of cell types in human ascending aorta also has been reported (Li et al., 2020). In this study, we found 9 types of cells with 20 clusters, including T cells, B cells, monocytes, macrophages, mast cells, neutrophils, SMCs, fibroblasts, and endothelial cells from 11 samples. We started with cluster analysis of T cells, reclustering, and subset analysis of T cells showed that the number of T cells decreased significantly in diseases, which weakened the defense mechanisms against disease injury. Some reports showed that immune cells were an important factor in causing a vascular rupture in AD development (Del Porto et al., 2010; Ju et al., 2013; Ye et al., 2018). In the subclusters of T cells, we found that the normal samples were mainly T helper/naive T cells, while in disease a, the C1 cells played a major role. T cells expressed a large number of heat shock protein genes, suggesting the cells might have oxidative stress damage and pro-inflammatory effects. Similar T cells have been reported in atherosclerosis (Fernandez et al., 2019). In disease b, T cells became macrophages that secreted chemokines that recruited inflammatory cells and promoted inflammatory responses. T cells were found to have pr-oinflammatory effects in Marfan syndrome (He et al., 2008; Wu et al., 2013b). According to GSVA, the inflammatory response of T cells in disease b was more intense than that in disease a, which may indicate that disease b has greater damage to vessels in AD.

VSMCs are the brick structures of blood vessels that maintain blood pressure and structural stability. Heterogeneity and variability of VSMCs have long been reported (Gomez and Owens, 2012; Bochaton-Piallat and Bäck, 2018; Frismantiene et al., 2018). Phenotypic plasticity of VSMCs has been reported in hypertension and Marfan-induced AD (Perrucci et al., 2017; Clément et al., 2019). Currently, single-cell sequencing has revealed the role of VSMC heterogeneity in diseases such as tumors and atherosclerosis (AS) (Cochain et al., 2018; Ding et al., 2020; Zhou et al., 2020; Williams et al., 2020). In our study, there were nine subgroups of VSMCs. In fact, contractile SMCs were the main components in both normal samples and disease vessels, but their functions were changed. Contractile VSMCs in Normal maintained the physiological function of blood vessels, while in AD, they promoted inflammation and regulated ECM. Most notably, SMCs differentiated into macrophage types in diseases, which promoted inflammatory responses. There were also differences between a and b. Disease a tended to regulate endothelial cell remodeling, while disease b had a more intense inflammatory response, secreted inflammatory factors, and increased the destruction of elastic fibers and collagen deposition. RNA velocity analysis suggested that disease a was actively differentiated and disease b was mainly differentiated towards inflammatory cells. GSVA further revealed the difference between diseases a and b. Disease a was related to IL-6 production, while disease b was associated with BMP and TGF-β pathways, which is consistent with the previous reports. Moreover, we found some new genes in diseases a and b. We also found some new genes that have not been reported in AD. CCL3L3 and CLMP, for example, could regulate inflammation reactions. CYR61 and LMOD1 are important genes for maintaining normal vascular structure. MGP inhibited vascular calcification and decreased in disease b, suggesting that disease b was more prone to calcification. TGM2 regulated angiogenesis and apoptosis in colorectal cancer (Yang et al., 2019), which is highly expressed in VSMCs of diseases, especially in disease a. Single-cell RNA sequencing explains the common and different mechanisms of hypertension and Marfan syndrome caused AD from different perspectives.

Progenitor cells in the vessel adventitia can transform into SMCs or ECs, contributing to organ and tissue fibrosis (Yu et al., 2018). Different subpopulations of vascular fibroblasts and other tissue and organ fibroblasts have been reported (Dobnikar et al., 2018), they may perform different functions in normal or diseases.

Our study discovered that the proportion of fibroblast subsets changed significantly in diseases suggesting fibroblasts may play a key role in the development of AD. Fibroblast clusters analysis showed that the differentiation direction of fibroblasts in the diseases was mainly SMCs or endothelial cells, which promoted inflammatory response. However, disease a mainly secreted a large number of collagen genes while disease b differentiated into contractible SMCs, which was helpful to maintain vascular function. In addition, normal fibroblasts originate from the spinal cord and have progenitor cells. The RNA velocity results suggested that fibroblasts and neural progenitor cells were more likely to differentiate. Disease a and b were transited to VSMCs. Moreover, FBN1/2 and ABIBP, important factors in maintaining vessels were expressed in normal, which reduced MMP-2 and oxidative stress damage, but decreased in diseases. In disease a, POSTN/TGFBI was also found to be highly expressed. BMP2, FOSL1, and HMGA1 genes were expressed in disease b. These genes were not reported in AD, which may be important factors in disease a and b induced AD.

Inflammation is ubiquitous and plays a crucial role in diseases, such as tumors or other diseases (Kim et al., 2013; Singh et al., 2019). Macrophages were mainly divided into M1 and M2 types in many studies. The studies on vascular macrophages were also mainly analyzed according to their location in the AS and ascending aorta (Xu et al., 2019; Li et al., 2020). Monocytes and macrophages were rearranged and divided into 12 subgroups. The cell proportion data showed that cells decreased significantly in diseases. Cellular differentiation revealed differences between diseases a and b, revealing similarities and differences in the pathogenesis of a and b. From this perspective, we found disease a was mainly manifested by the inflammatory response and tissue remodeling processes of macrophages, while disease b was more significant in the destruction of fibrous tissues and collagen changes.

Extensive communication between tumor cells was found by single-cell RNA sequencing (Carystinos et al., 2001; Hale et al., 2012; Kumar et al., 2018), and significant interaction between macrophages and T cells was reported in COVID-19 (He et al., 2021). It indicated that intercellular communication plays an important role in diseases. Macrophages communicate with other cells widely, such as in cancer and AS (Dehne et al., 2017; Pajarinen et al., 2019). We also found that interactions between macrophages and T cells, as well as SMCs and fibroblasts, promoted phenotypic transformation and inflammation in the diseases. In addition, fibroblasts and SMCs have a strong influence on other cells during diseases and play an important role in promoting cell differentiation and vascular remodeling, indicating that the occurrence of diseases is caused by the complex network of multiple cells.

In conclusion, we used a single cell RNA sequence to identify subtle communication among T cells, SMCs, fibroblasts, and Mo-Mac subpopulations in hypertension and Marfan-induced AD. These new insights into AD progression may be useful for a better understanding of the different causes, which may identify interactions that are predictive biomarkers of response to prevention for AD.

## Data Availability

The original contributions presented in the study are publicly available. This data can be found here: (http:www.ncbi.nlm.nih.gov/bioproject/847544).
