# Supplementary material for: Single Cell RNA Sequencing Reveals the Pathogenesis of Aortic Dissection Caused by Hypertension and Marfan Syndrome
Source: Front Cell Dev Biol. 2022 Jun 21;10:880320. doi: 10.3389/fcell.2022.880320 (PMC9253298; doi:10.3389/fcell.2022.880320)

SUPPLEMENTAL MATERIAL

Single cell RNA sequencing reveals the pathogenesis of aortic dissection caused by hypertension and Marfan syndrome

Li Zhang[1,2i], Zhihuang Qiu[1i], Hui Zheng[1], Xi Yang[1, 3], Yameng Zheng[2], Jianqiang Ye[3], Jian He[1],Yumei Li[1, 3], Liangwan Chen[1]

[1]Department of Cardiac Surgery, Fujian Medical University Union Hospital, [2]The Key Laboratory of Fujian Province Universities on Ion Channel and Signal Transduction in Cardiovascular Diseases, The School of Basic Medical Sciences, [3]Fujian Center for Safety Evaluation of New Drug, Fujian Medical University, Fuzhou, China, and [4]Novogene Co, Ltd, Beijing, China.

[i] The co-authors.

*Correspondence author: Liangwan Chen and Yumei Li, Fujian Medical University Union Hospital, NO 29 XinQian Road, Fuzhou,Fuzhou, 350001, Fujian, China.

Tel: +86-591-83301393; Fax: +86-591-83344034; Email:chenliangwan@fjmu.edu.cn.

Supplemental Table 1. T cell GSVA （A vs. N）

Pathways	logFC	AveExpr	t	P.Value	adj.P.Val	B
PERK-mediated unfolded protein response	-0.17476	-0.39817	-24.3888	########	########	275.1095
inflammatory response	-0.03914	-0.32338	-19.6378	2.92E-83	3.36E-82	178.4731

cytokine-mediated signaling pathway	-0.04508	-0.32789	-19.5106	3.05E-82	2.34E-81	176.1347
positive regulation of angiogenesis	-0.04092	-0.2965	-17.2273	5.97E-65	3.43E-64	136.4553
positive regulation of interferon-gamma production	-0.0428	-0.39756	-11.5414	1.71E-30	7.87E-30	57.53299
positive regulation of epithelial to mesenchymal transition	-0.03024	-0.45151	-10.1024	8.36E-24	3.20E-23	42.26665
interferon-gamma-mediated signaling pathway	-0.03215	-0.35894	-9.44988	4.75E-21	1.56E-20	35.99056
cell cycle arrest	-0.02327	-0.40666	-9.15554	7.28E-20	2.09E-19	33.29259
positive regulation of protein phosphorylation	-0.01929	-0.34739	-9.08583	1.37E-19	3.51E-19	32.66578
positive regulation of NF-kappaB transcription factor activity	-0.01799	-0.40095	-8.55898	1.43E-17	3.29E-17	28.0793
Fc-gamma receptor signaling pathway involved in phagocytosis	0.025238	-0.19547	8.462352	3.26E-17	6.82E-17	27.26714
angiogenesis	-0.01519	-0.30494	-7.54903	5.04E-14	9.66E-14	20.03613
positive regulation of cell migration	-0.0151	-0.3504	-7.16961	8.43E-13	1.49E-12	17.2701
cellular response to oxidative stress	-0.01758	-0.40363	-6.28437	3.52E-10	5.79E-10	11.36248
positive regulation of vascular smooth muscle cell proliferation	0.023976	-0.39001	6.111541	1.05E-09	1.61E-09	10.29861
integrin-mediated signaling pathway	-0.01726	-0.34441	-5.55042	2.97E-08	4.27E-08	7.046649
neutrophil degranulation	-0.01097	-0.3809	-3.88868	0.000102	0.000138	-0.76274
negative regulation of apoptotic process	-0.00896	-0.35152	-3.79321	0.00015	0.000192	-1.12838
tumor necrosis factor-mediated signaling pathway	0.009974	-0.44484	3.474305	0.000516	0.000624	-2.28416
positive regulation of gene expression	-0.00397	-0.32011	-2.45487	0.014122	0.01624	-5.30052
extracellular matrix organization	0.004338	-0.2674	2.251613	0.024383	0.026705	-5.77818
immune response	0.002815	-0.19578	1.300911	0.193339	0.202127	-7.46521
extracellular matrix disassembly	-0.00013	-0.29713	-0.04205	0.966463	0.966463	-8.31003

Supplemental Table 2. T cell GSVA （B vs. N）

Pathways	logFC	AveExpr	t	P.Value	adj.P.Val	B
viral transcription	0.257647	-0.02038	22.97766	########	########	242.7222

translational initiation	0.190462	-0.23559	22.95604	########	########	242.273
SRP-dependent cotranslational protein targeting to membrane	0.288271	-0.03224	22.92492	########	########	241.6273
translation	0.166649	-0.2151	22.87403	########	########	240.5728
immune response	0.045676	-0.22637	16.14066	3.54E-57	1.42E-56	118.7869
leukocyte migration	0.043068	-0.21512	14.72425	4.46E-48	1.49E-47	97.93358
Fc-gamma receptor signaling pathway involved in phagocytosis	0.04867	-0.19877	13.10202	1.37E-38	3.90E-38	76.21782
positive regulation of cell proliferation	0.026189	-0.30999	12.42015	6.58E-35	1.64E-34	67.79553
innate immune response	0.027161	-0.36429	10.90025	2.32E-27	5.16E-27	50.55364
positive regulation of NF-kappaB transcription factor activity	0.021475	-0.40184	7.998276	1.55E-15	3.11E-15	23.63977
interleukin-1-mediated signaling pathway	0.027671	-0.45637	7.359018	2.15E-13	3.91E-13	18.7914
neutrophil degranulation	0.024796	-0.37633	7.12594	1.18E-12	1.97E-12	17.1219
inflammatory response	-0.01079	-0.32103	-4.33514	1.49E-05	2.28E-05	1.249589
positive regulation of vascular endothelial growth factor production	-0.01952	-0.30241	-4.26224	2.06E-05	2.94E-05	0.937555
extracellular matrix disassembly	0.01467	-0.28925	3.918227	9.04E-05	0.000121	-0.46391
integrin-mediated signaling pathway	0.009362	-0.3545	2.526228	0.01156	0.01445	-4.93659
cytokine-mediated signaling pathway	0.005004	-0.31837	1.719641	0.085559	0.100658	-6.6462
extracellular matrix organization	-0.00339	-0.27136	-1.32252	0.186055	0.206728	-7.24957
positive regulation of smooth muscle cell proliferation	-0.00227	-0.35	-0.63754	0.5238	0.551368	-7.92028
positive regulation of angiogenesis	-0.00031	-0.33606	-0.09933	0.920877	0.920877	-8.11844

Supplemental Table 2. T cell GSVA （A vs. B）

Pathways	logFC	AveExpr	t	P.Value	adj.P.Val	B
viral transcription	-0.17222	-0.02954	-14.381	1.76E-45	2.81E-44	92.1909
nuclear-transcribed mRNA catabolic process, nonsense-mediated decay	-0.13159	-0.22207	-14.2263	1.42E-44	1.14E-43	90.11382

SRP-dependent cotranslational protein targeting to membrane	-0.18414	-0.05208	-14.0624	1.27E-43	6.79E-43	87.9346
translation	-0.10924	-0.21858	-13.9923	3.23E-43	1.29E-42	87.01048
negative regulation of transforming growth factor beta receptor signaling pathway	-0.05135	-0.36964	-13.8995	1.10E-42	3.52E-42	85.79121
interferon-gamma-mediated signaling pathway	-0.06244	-0.3559	-13.7734	5.75E-42	1.53E-41	84.14837
response to unfolded protein	-0.12383	-0.22469	-13.7555	7.26E-42	1.66E-41	83.9156
negative regulation of transcription by RNA polymerase II	-0.03444	-0.3749	-13.5516	1.02E-40	2.04E-40	81.28848
elastic fiber assembly	0.048806	-0.16306	12.89238	4.15E-37	7.38E-37	73.03614
positive regulation of NF-kappaB transcription factor activity	-0.03671	-0.38167	-12.6805	5.54E-36	8.87E-36	70.46205
protein stabilization	-0.04476	-0.37328	-12.2986	5.38E-34	7.83E-34	65.92047
response to virus	-0.05315	-0.30884	-11.0773	5.22E-28	6.95E-28	52.25092
transforming growth factor beta receptor signaling pathway	-0.0241	-0.42037	-8.64856	8.13E-18	1.00E-17	29.04233
innate immune response	-0.02208	-0.37021	-7.87083	4.79E-15	5.47E-15	22.76059
osteoblast differentiation	-0.02563	-0.29162	-6.61305	4.40E-11	4.69E-11	13.81038
angiogenesis	-0.01313	-0.25134	-5.1646	2.56E-07	2.56E-07	5.383217

SUPPLEMENTAL MATERIAL

Single cell RNA sequencing reveals the pathogenesis of aortic dissection caused by hypertension and Marfan syndrome

Li Zhang[1,2i], Zhihuang Qiu[1i], Hui Zheng[1], Xi Yang[1, 3], Yameng Zheng[2], Jianqiang Ye[3], Jian He[1],Yumei Li[1, 3], Liangwan Chen[1]

[1]Department of Cardiac Surgery, Fujian Medical University Union Hospital, [2]The Key Laboratory of Fujian Province Universities on Ion Channel and Signal Transduction in Cardiovascular Diseases, The School of Basic Medical Sciences, [3]Fujian Center for Safety Evaluation of New Drug, Fujian Medical University, Fuzhou, China, and [4]Novogene Co, Ltd, Beijing, China.

[i] The co-authors.

*Correspondence author: Liangwan Chen and Yumei Li, Fujian Medical University Union Hospital, NO 29 XinQian Road, Fuzhou,Fuzhou, 350001, Fujian, China.

Tel: +86-591-83301393; Fax: +86-591-83344034; Email:chenliangwan@fjmu.edu.cn.

Supplemental Figure 1. The quality control of the cells.

A. Cell selection standard for all samples.

B. The cell number and reads of each sample.

C. The total UMAP plots of all samples.

D. The cell normalization and cell filtering considering the MT percentage, minimum and maximum gene numbers by the Seurat [https://satijalab.org/seurat/]

package.

E. Umi-tools was applied for Single Cell Transcriptome Analysis to identify the cell barcode whitelist, extract the cell barcode UMIs and Calculate the cell expression counts based on the filtered clean fastq data.

F. The UMAP plots of each sample.

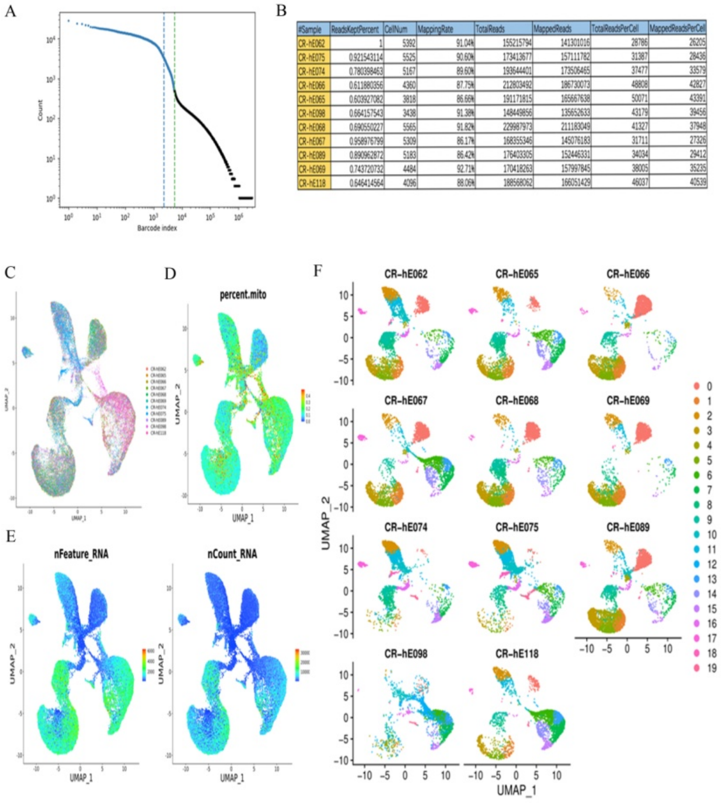

#Sample	ReadsKeptPercent	CellNum	MappingRate	TotalReads	MappedReads	TotalReadsPerCell	MappedReadsPerCell
CR-hE062	1	5392	91.04%	155215794	141301016	28786	26205
CR-hE075	0.921543114	5525	90.60%	173413677	157111782	31387	28436
CR-hE074	0.780398463	5167	89.60%	193644401	173506465	37477	33579
CR-hE066	0.611880356	4360	87.75%	212803492	186730073	48808	42827
CR-hE065	0.603927082	3818	86.66%	191171815	165667638	50071	43391
CR-hE098	0.664157543	3438	91.38%	148449856	135652633	43179	39456
CR-hE068	0.690550227	5565	91.82%	229987973	211183049	41327	37948
CR-hE067	0.958976799	5309	86.17%	168355346	145076183	31711	27326
CR-hE089	0.890962872	5183	86.42%	176403305	152446331	34034	29412
CR-hE069	0.743720732	4484	92.71%	170418263	157997845	38005	35235
CR-hE118	0.646414564	4096	88.06%	188568062	166051429	46037	40539

Supplemental Figure 2. Clustering analysis for all cells.

A. Genes expression heatmaps for integrated analysis.

B. The heatmap of enriched pathways in each cluster (C0~19).

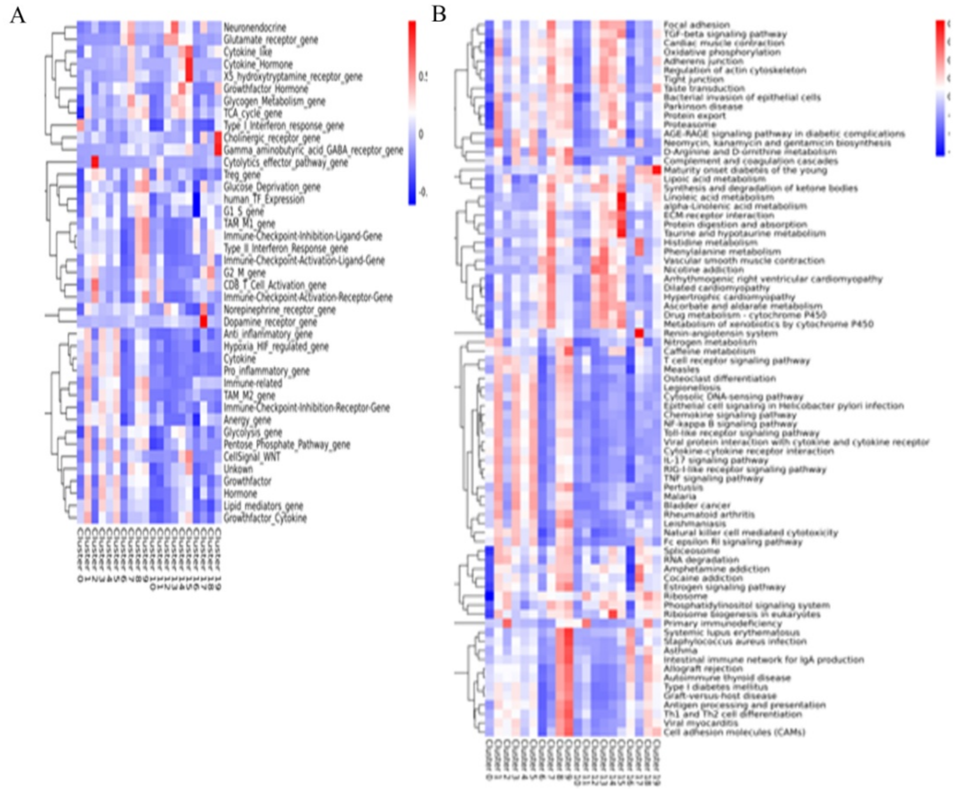

Supplemental Figure 3. Clustering analysis for T cells.

A. Differential gene expression level sequencing for each subset.

B. Top genes in each cluster.

C.The different pathways between A vs. N, B vs. N and A vs. B by GO enrichment analysis.

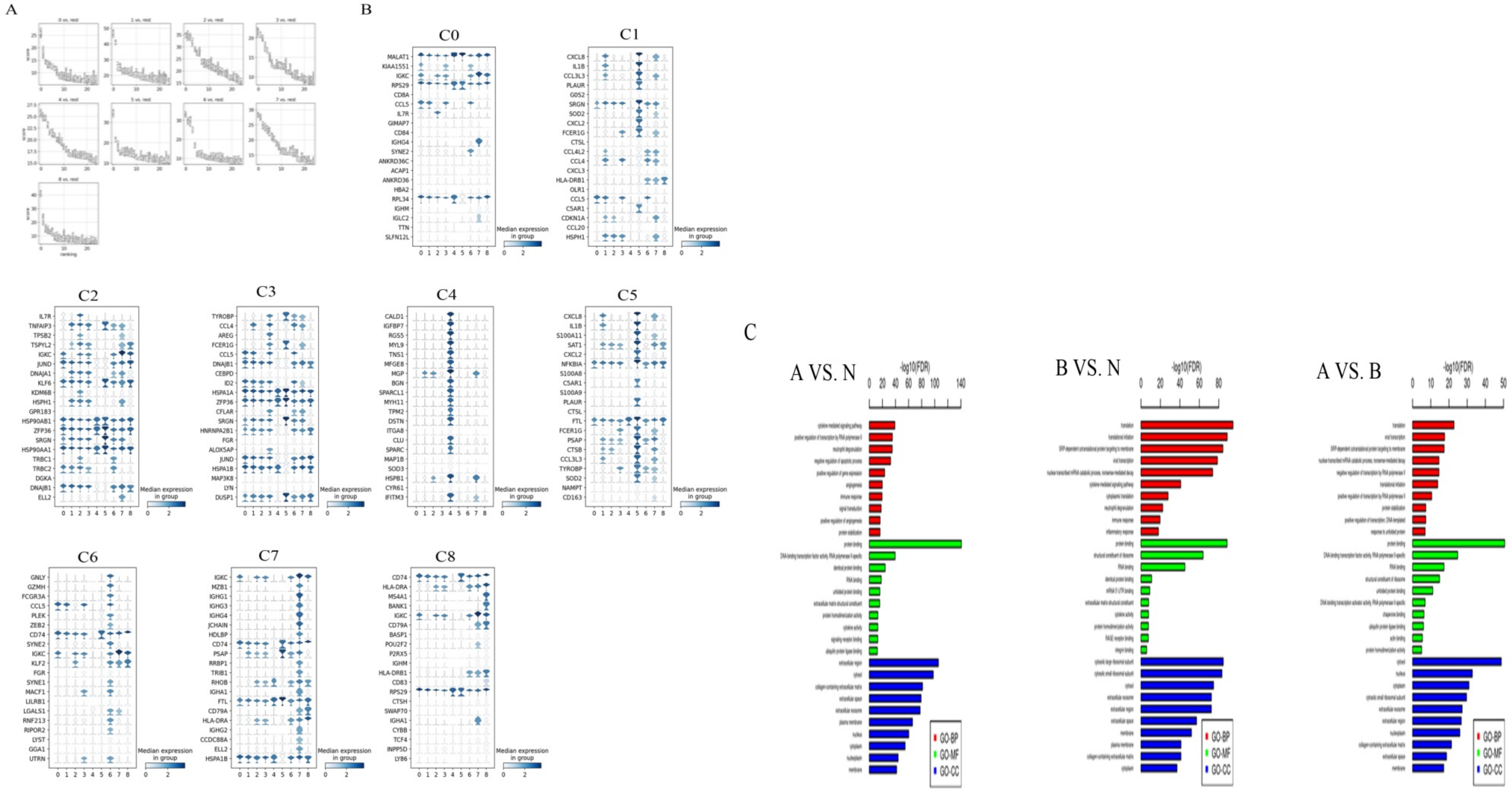

Supplemental Figure 4. Clustering analysis for SMCs.

A. Differential gene expression level sequencing for each subset.

B. Top genes in each cluster.

C. GO enrichment pathways in each cluster.

D. The violin diagram shows the changed genes in diseases.

E. GSVA analysis showed the changed pathways in each sub-cluster of SMCs.

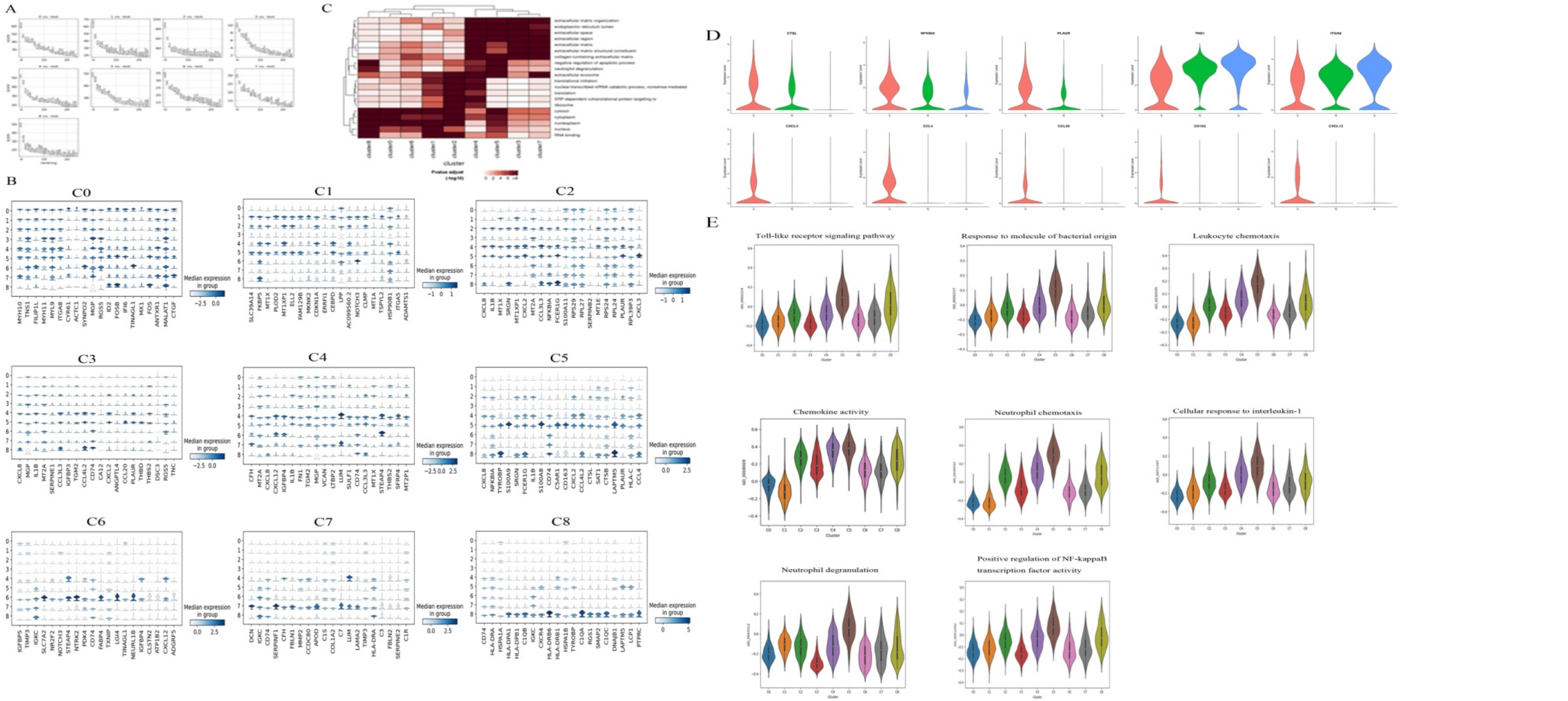

Supplemental Figure 5. Clustering analysis for fibroblasts.

A. Top genes in each cluster as shown in UMAP plots.

B. Top genes in each cluster as shown in violin plots.

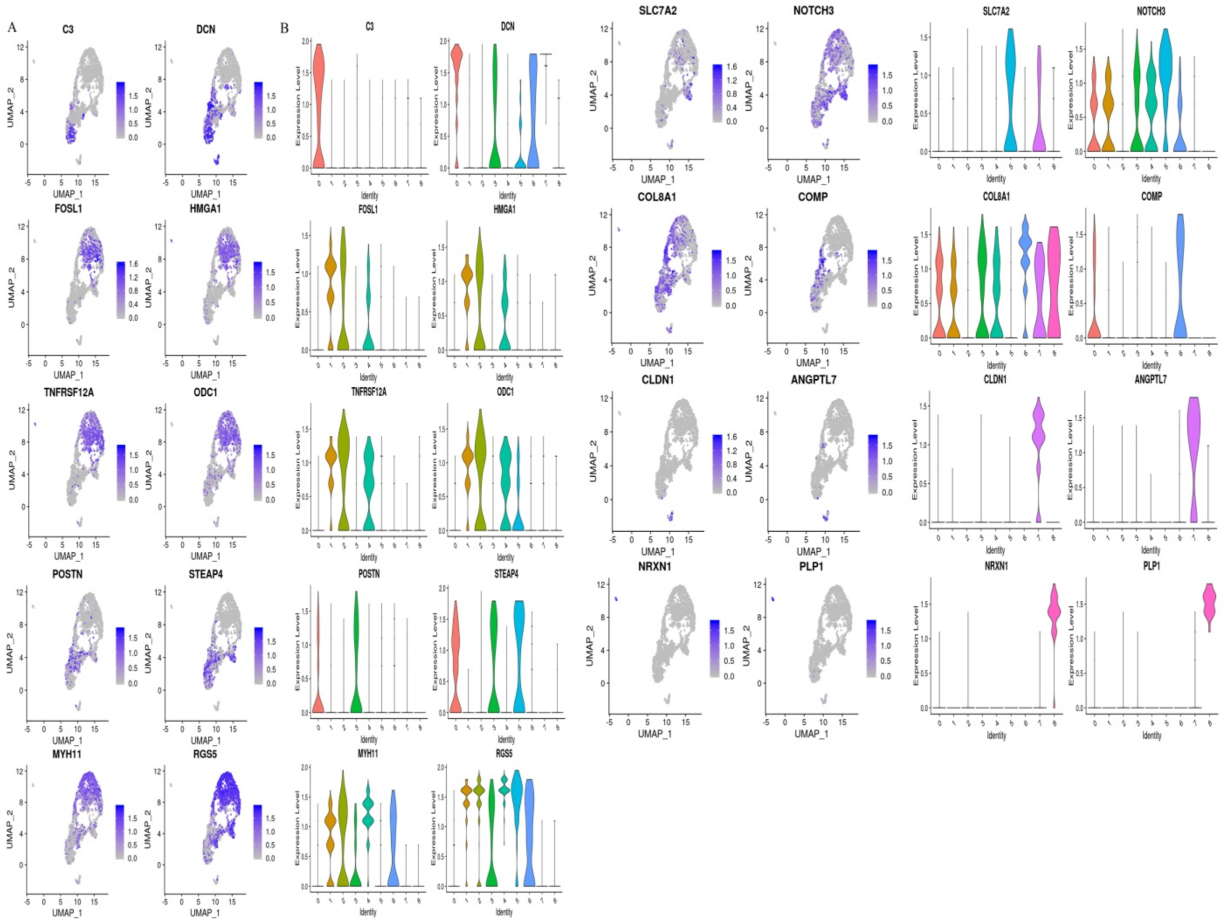

Supplemental Figure 6. Cell-cell communication.

A. Circular network diagram of important cell-cell interaction pathways. Arrow and edge color represent direction (ligand and receptor), edge thickness represents the sum of weighted paths among sub-clusters of T, SMC, fibroblast and Mo-Mac cells.

B. The dot plot shows the key ligands in the outing and incoming signal mode of the subset of secretory cells and receiver cells.

C. Heatmap shows the outgoing and incoming communication patterns calculated by the key signals for subpopulations of target cells.

D. River map shows the outgoing and incoming communication patterns of target cells by the key signals for subpopulations.

E-L. Hierarchical network diagram of important cell-cell communication modes among Tcell, SMC, Fibroblast and Mo-Mac. Edge thickness represents the sum of key signals of weight between subgroups.

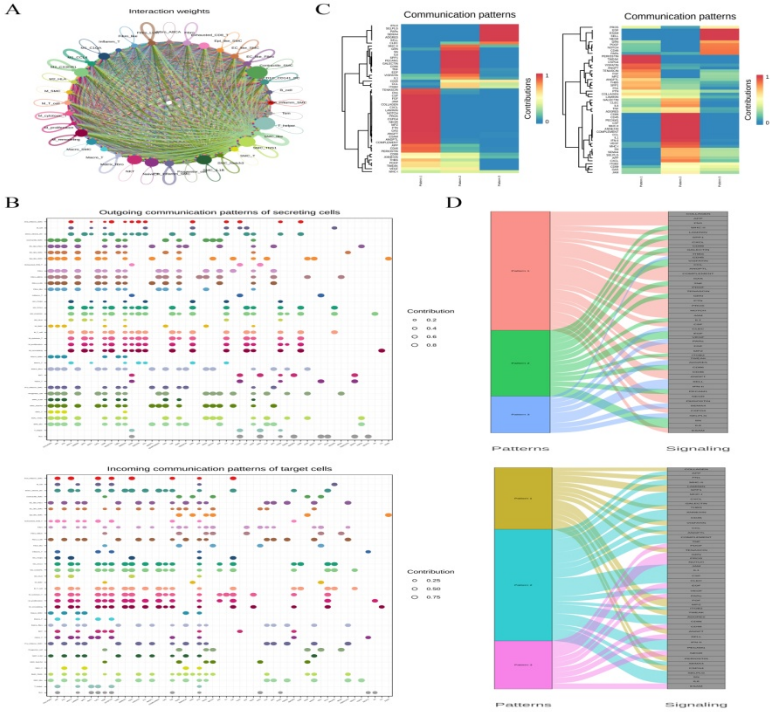

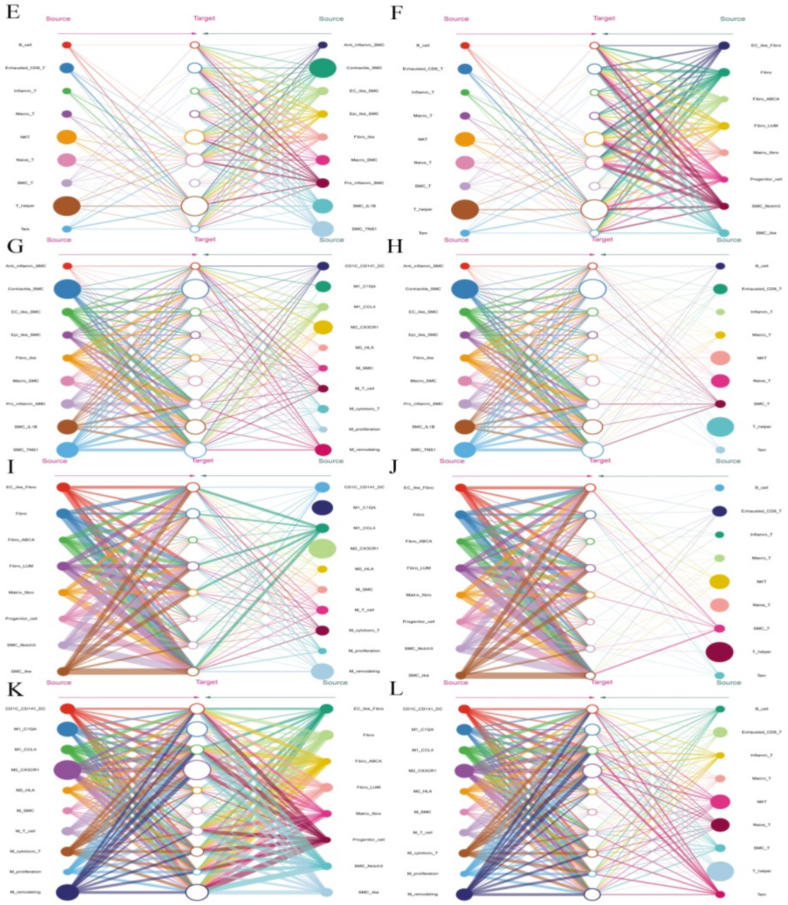

Supplement: Supplementary file 1 [file DataSheet1.PDF]
